# Biopsy-proven tyrosine kinase inhibitor–associated renal injury: a case series

**DOI:** 10.1093/ckj/sfaf394

**Published:** 2025-12-13

**Authors:** Jingying Lian, Jing Tian, Shaoshan Liang, Feng Xu, Fan Yang, Dacheng Chen, Xiaodong Zhu, Yongzhong Zhong, Caihong Zeng

**Affiliations:** National Clinical Research Center for Kidney Diseases, Jinling Clinical Medical College, Nanjing Medical University, Nanjing, China; National Clinical Research Center for Kidney Diseases, Jinling Hospital, Affiliated Hospital of Medical School, Nanjing University, Nanjing, China; National Clinical Research Center for Kidney Diseases, Jinling Hospital, Affiliated Hospital of Medical School, Nanjing University, Nanjing, China; National Clinical Research Center for Kidney Diseases, Jinling Hospital, Affiliated Hospital of Medical School, Nanjing University, Nanjing, China; National Clinical Research Center for Kidney Diseases, Jinling Hospital, Affiliated Hospital of Medical School, Nanjing University, Nanjing, China; National Clinical Research Center for Kidney Diseases, Jinling Hospital, Affiliated Hospital of Medical School, Nanjing University, Nanjing, China; National Clinical Research Center for Kidney Diseases, Jinling Hospital, Affiliated Hospital of Medical School, Nanjing University, Nanjing, China; National Clinical Research Center for Kidney Diseases, Jinling Hospital, Affiliated Hospital of Medical School, Nanjing University, Nanjing, China; National Clinical Research Center for Kidney Diseases, Jinling Clinical Medical College, Nanjing Medical University, Nanjing, China

**Keywords:** albuminuria, kidney biopsy, nephrotoxicity, thrombotic microangiopathy, VEGF

## Abstract

**Background:**

Tyrosine kinase inhibitors (TKIs) are essential anticancer agents associated with substantial nephrotoxic potential. Although TKI-induced renal injury is increasingly recognized, comprehensive histopathological characterization remains limited due to insufficient renal biopsy data. This study characterizes the clinicopathological spectrum and outcomes of biopsy-proven TKI nephrotoxicity.

**Methods:**

This retrospective study analyzed 21 patients with biopsy-proven TKI-associated renal injury identified between 2015 and 2025. Demographic characteristics, renal function indices, oncological profiles and histopathological features were analyzed.

**Results:**

The cohort included 16 patients with solid tumors and 5 with hematologic malignancies exposed to four major TKI classes: vascular endothelial growth factor receptor, platelet-derived growth factor receptor, human epidermal growth factor receptor and Bruton’s tyrosine kinase TKIs. The median time from TKI initiation to symptom onset was 9.5 months. Clinical manifestations included proteinuria (95%), edema (52%) and new-onset/worsened hypertension (47%). At biopsy, median serum creatinine was 1.07 mg/dL (94.6 µmol/L) and proteinuria was 1.83 g/day. Histopathological analysis demonstrated thrombotic microangiopathy (TMA)-like lesions in 17 of 21 cases (80%), with concurrent immunoglobulin A nephropathy in 3 cases and focal segmental glomerulosclerosis in 3 cases. Among 18 patients with available follow-up data, 14 discontinued their initial TKI therapy, with 5 transitioning to alternative TKIs. Treatment strategies included angiotensin-converting enzyme inhibitor/angiotensin-receptor blocker monotherapy (*n* = 13) and combination therapy with corticosteroids/immunosuppressants (*n* = 5). During a median follow-up period of 9.5 months, complete and partial proteinuria remission occurred in five cases each. Four patients died due to cancer progression, while renal function remained stable in the remaining patients without progression to end-stage renal disease.

**Conclusion:**

TKI-induced renal injury characteristically presents with edema, hypertension and significant proteinuria, with renal-limited TMA as the predominant histopathological finding. Timely recognition and prompt discontinuation of the offending TKI, coupled with appropriate supportive nephroprotective management, generally yield favorable long-term renal outcomes with preservation of kidney function.

## GRAPHICAL ABSTRACT

**GRAPHICAL ABSTRACT:**
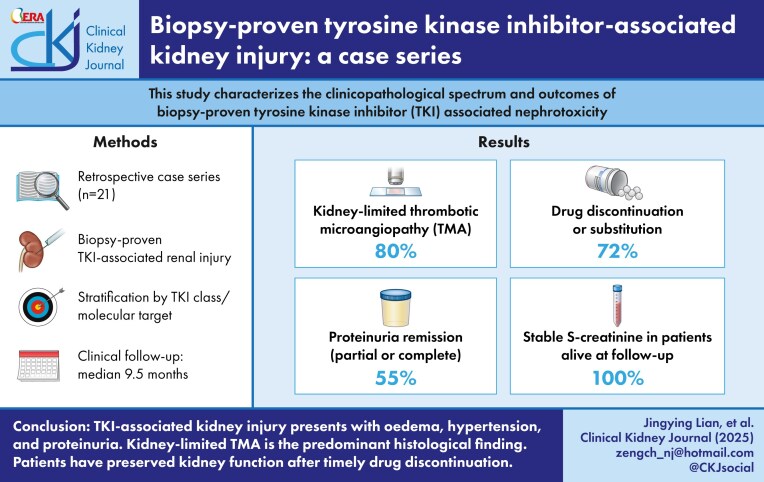

KEY LEARNING POINTS **What was known:**

- Tyrosine kinase inhibitors (TKIs) have become essential components of anticancer therapy.
- TKIs cause significant nephrotoxicity, notably proteinuria, with incidence rates of 11.7%–18.7%.
- Comprehensive histopathological characterization has been limited by insufficient renal biopsy data.

**This study adds:**

- TKI-induced renal injury characteristically presents with edema, hypertension and significant proteinuria, with renal-limited thrombotic microangiopathy (TMA) as the predominant histopathological finding.
- Timely recognition and prompt discontinuation of the offending TKI, coupled with appropriate supportive nephroprotective management, generally yield favorable long-term renal outcomes with preservation of kidney function.

**Potential impact:**

- This study establishes renal TMA as a cardinal pathologic mechanism of TKI nephrotoxicity; this finding provides a definitive diagnostic clue for clinicians.
- Our outcomes demonstrate that timely intervention, primarily through TKI discontinuation, effectively prevents irreversible renal failure, thereby securing quality oncologic care without compromising long-term kidney health.

## INTRODUCTION

Since the US Food and Drug Administration first approved imatinib for chronic myeloid leukemia in 2001, tyrosine kinase inhibitors (TKIs) have become essential components of

anticancer therapy. Currently, over 70 distinct TKI agents have received clinical approval, targeting 17 critical signaling pathways, including vascular endothelial growth factor receptor (VEGFR), human epidermal growth factor receptor (HER), platelet-derived growth factor receptor (PDGFR) and Bruton’s tyrosine kinase (BTK). These agents demonstrate substantial efficacy against both hematologic malignancies and solid tumors, with several TKIs now established as standard first-line options, underscoring their pivotal role in contemporary oncology.

However, TKIs are associated with significant adverse effect profiles, particularly drug-induced nephrotoxicity. Clinical trials and real-world data reveal proteinuria incidence rates ranging from 11.7% to 18.7% [1, 2], frequently requiring dose modifications or treatment discontinuation which can compromise therapeutic outcomes and increase healthcare costs. Although reports of TKI-related renal injury continue to accumulate, comprehensive histopathological characterization has been limited by insufficient renal biopsy data. Therefore, we conducted a retrospective analysis of biopsy-confirmed TKI-associated renal injury to systematically characterize the clinical manifestations, histopathological features and prognostic outcomes of this emerging nephropathy.

## MATERIALS AND METHODS

### Patients and clinical data

This retrospective cohort study included patients diagnosed with TKI-associated renal injury confirmed by renal biopsy at the National Clinical Research Center for Kidney Diseases between January 2015 and March 2025 inclusive. Inclusion criteria were: (i) confirmed TKI exposure antecedent to renal injury onset; (ii) histopathologically confirmed renal lesions; and (iii) drug causality assessment graded as possible, probable or certain per World Health Organization–Uppsala Monitoring Centre (WHO-UMC) criteria for adverse drug reactions. Exclusion criteria were: (i) exposure to nephrotoxic agents distinct from antitumor therapies; (ii) renal biopsy specimens containing fewer than five glomeruli; and (iii) renal transplant recipients. The study protocol was approved by the Ethics Committee of Jinling Hospital (2024DZKY-083-01).

Demographic and clinical parameters were extracted from electronic medical records, including sex, age, underlying malignancy type, TKI regimen, treatment duration, concomitant medications and renal function indices at the time of renal biopsy. Clinical manifestations, including edema, hypertension and hematuria, were systematically recorded. Follow-up data included post-biopsy therapeutic interventions, serial assessments of serum creatinine and proteinuria, along with oncological outcomes.

Acute kidney injury (AKI) was diagnosed and staged according to the Kidney Disease: Improving Global Outcomes (KDIGO) clinical practice guidelines [3]. Nephrotic-range proteinuria was defined as ≥3.5 g/24 h, hypoalbuminemia as serum albumin ≤30 g/L and nephrotic syndrome by concurrent manifestation of both. Microscopic hematuria required >10⁵ RBCs/mL (100/μL) in urinary sediment, while renal insufficiency was characterized by serum creatinine >1.24 mg/dL (>110 µmol/L). Serum creatinine trajectory was stratified into: recovery was defined as a decline in serum creatinine by ≥25% or ≥0.3 mg/dL (≥26.5 µmol/L) from the diagnosis-level value; stability was defined as a change in serum creatinine of <25% and <0.3 mg/dL (<26.5 µmol/L) from the diagnosis-level value; and worsening was defined as an increase in serum creatinine by ≥25% or ≥0.3 mg/dL (≥26.5 µmol/L) from the diagnosis-level value. Proteinuria remission was stratified into: complete (CR: <0.4 g/24 h or urine protein-to-creatinine ratio <300 mg/g with preserved renal function), partial (PR: ≥50% reduction from the diagnosis-level value to <3.5 g/24 h) or no remission (failure to achieve CR/PR criteria. Renal survival was staged per KDIGO guidelines, defining ESRD as eGFR <15 mL/min/1.73 m². The eGFR was derived using the Chronic Kidney Disease Epidemiology Collaboration equation. Tumor status assessment adhered to Response Evaluation Criteria in Solid Tumors (RECIST) 1.1 [4] (solid tumors), European LeukemiaNet (ELN) [5] (leukemias) and Lugano [6] criteria (lymphomas).

### Pathology studies

All renal biopsies were processed using the standard techniques of light microscopy, immunofluorescence and electron microscopy. Light microscopic tissues were paraffin-embedded, sectioned at 2 μm, and stained with hematoxylin and eosin, periodic acid–Schiff (PAS), periodic acid–Schiff methenamine (PASM)-Masson and Masson trichrome. Immunofluorescence frozen tissue sections of 3 μm were stained with immunoglobulin G (IgG), IgA, IgM, C3 and C1q (direct method, Dako). Immunofluorescence staining intensity was graded on a scale of 0 to +++. On light microscope, acute tubular injury, tubular atrophy and interstitial fibrosis were graded on a semiquantitative scale based on an estimate of the percentage of renal cortex affected and recorded as – (0), + (1%–25%), ++ (25%–50%) or +++ (>50%). Ultrastructural evaluation was performed using a FEI Tecnai G2 Spirit transmission electron microscope.

### Statistical methods

Statistical analyses were conducted using SPSS Statistics 26.0 (IBM Corp., Armonk, NY, USA). Continuous variables conforming to normal distribution were presented as mean ± standard deviation; non-normally distributed continuous variables as median with interquartile range (IQR); and categorical variables as frequencies and percentages (%).

## RESULTS

### Causality assessment

Given the complexity of polypharmacy in cancer patients, we systematically assessed TKI-related nephrotoxicity causality in accordance with WHO-UMC criteria [7]. Among the 21 biopsy-confirmed cases, causality was classified as: certain (*n* = 1), probable (*n* = 18) and possible (*n* = 2). The two cases classified as possible (Cases 5 and 19) were retained for analysis despite the presence of confounding medications.

### Demographic and clinical characteristics

**Figure 1:**
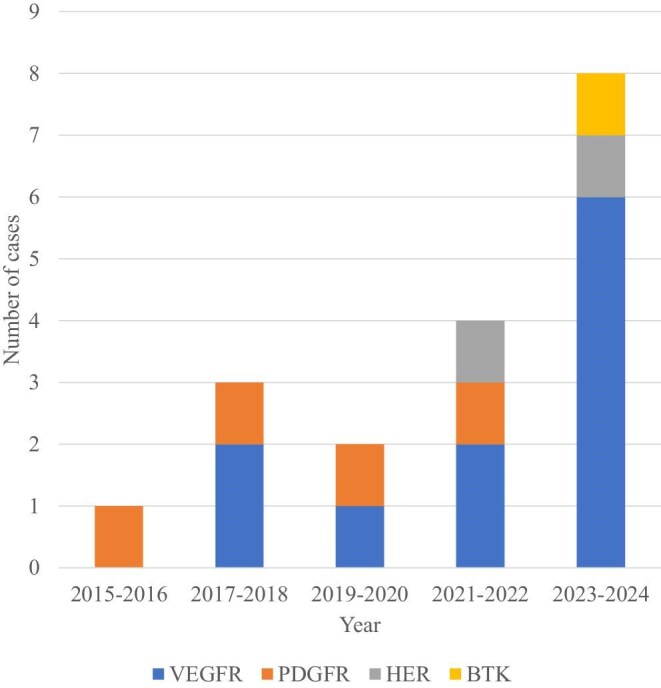
Number of Cases of TKI-associated renal injury with different target class (2015–24).

The cohort included 16 patients with solid tumors (9 gastrointestinal, 5 respiratory, 1 thyroid, 1 breast) and 5 with hematologic malignancies (4 chronic myeloid leukemia, 1 mantle cell lymphoma), as detailed in Table 1. Figure 1 demonstrates the increasing incidence of biopsy-confirmed TKI-associated renal injury cases from 2015 to 2024. Patients received 15 distinct TKIs targeting four principal signaling pathways: VEGFR inhibitors (anlotinib, lenvatinib, sorafenib, sunitinib, apatinib, fruquintinib, regorafenib); PDGFR inhibitors (dasatinib, imatinib, nilotinib, ripretinib); HER antagonists (aumolertinib, pyrotinib, osimertinib); and BTK inhibitors (zanubrutinib). Twelve patients had received prior anticancer therapy.

**Table 1:** Oncological profiles of 21 patients with TKI-associated renal injury: stratification by serum creatinine trajectory.

Pt	Underlying cancer	TKI	Main target	Concomitant medication	TKI dur. (mos)	Tumor status at biopsy	Renal biopsy findings
Recovery group							
1	Liver	Sorafenib	VEGFR	NA	105	CR	TMA
2	GIST	Sunitinib	VEGFR	NA	24	SD	TMA + IgAN
3	Lung	Ametinib	HER	NA	5.75	PD	IgAN + ATIN
Stability group							
4	Lung	Anlotinib	VEGFR	DTX	1	SD	TMA
5	Lung	Anlotinib	VEGFR	BEV	0.8	PR	TMA
6	Lung	Anlotinib	VEGFR	PEM, PTX, Plat.	0.63	PD	TMA
7	Thyroid	Lenvatinib	VEGFR	NA	53.4	PD	TMA + IgAN
8	Liver	Lenvatinib	VEGFR	NA	18	SD	TMA + FSGS
9	stomach	Apatinib	VEGFR	NA	53	SD	IgAN + TMA
10	GIST	Regorafenib	VEGFR	NA	30	PD	TMA
11	GIST	Sunitinib	VEGFR	NA	26	SD	TMA
12	CML	Dasatinib	PDGFR	NA	59	SD	TMA
13	Breast	Pyrotinib	HER	NA	34	SD	TMA
14	MCL	Zanubrutinib	BTK	RTX	5	SD	ATIN + ICG
Worsening group							
15	CML	Nilotinib	PDGFR	NA	36	SD	MN
Not assessable							
16	Colon	Fruquintinib	VEGFR	NA	1	SD	TMA
17	Liver	Lenvatinib	VEGFR	TDF	41	PD	TMA + FSGS
18	CML	Imatinib	PDGFR	NA	0.5	SD	TMA
19	CML	Dasatinib	PDGFR	NA	56	SD	TMA
20	GIST	Ripretinib	PDGFR	NA	25	SD	TMA + FSGS
21	Lung	Osimertinib	HER	NA	14	SD	CTIN

BEV, bevacizumab; CML, chronic myeloid leukemia; CTIN, chronic tubulointerstitial nephritis; Dur., duration; DTX, docetaxel; GIST, gastrointestinal stromal tumor; ICG, immune complex glomerulonephritis; MCL, mantle cell lymphoma; MN, membranous nephropathy; mos, months; NA, not available; PD, progressive disease; PEM, pemetrexed; Plat., platinum-based drugs; Pt, patient; SD, stable disease; TDF, tenofovir disoproxil.

Patient demographic and clinical characteristics are summarized in Tables 2 and 3. At the time of renal biopsy, the cohort had a mean age of 54.6 ± 8.8 years with male predominance (13/21, 61.9%). Eight patients (38.1%) had pre-existing renal comorbidities: isolated urinary abnormalities (*n* = 4), elevated serum creatinine (*n* = 3) or combined manifestations (*n* = 1)—all of whom experienced disease progression post-TKI initiation. The median time interval from TKI commencement to symptom onset was 9.5 months. Predominant clinical features included edema (52.4%), new-onset or worsening hypertension (47.6%) and nephrotic syndrome (38.1%). AKI was observed in only one patient (4.8%), with onset shortly before TKI initiation. In this case, renal dysfunction persisted and progressed following TKI administration, despite discontinuation of other potential causative agents. The median serum creatinine at biopsy was 1.07 mg/dL (94.6 µmol/L), with 42.9% (9/21) exceeding 1.24 mg/dL (110 µmol/L). Peak proteinuria levels reached a median of 3.93 g/24 h before biopsy, declining to 1.83 g/24 h at the time of renal biopsy. Notably, three patients (14.3%) maintained nephrotic-range proteinuria during biopsy evaluation. Laboratory abnormalities included anemia (66.7%), microscopic hematuria (52.4%), hypoalbuminemia (42.9%), hypocomplementemia (35.0%) and thrombocytopenia (9.5%). Gross hematuria was universally absent. Elevated lactate dehydrogenase was detected in seven patients (33.3%). Peripheral blood smears analyzed for schistocytes in five cases were negative.

**Table 2:** Clinical characteristics and laboratory parameters of TKI-associated renal injury by target type.

Group	TKI dur. (mos)	AKI	NS	SCr mg/dL (μmol/L)	U-Pro (g/day)	Alb (g/L)	Hb (g/L)	TCP
VEGFR (*n* = 12)	25 (1–47)	0	7/12	1.0 (0.6–1.2) [84 (56–108)]	1.83 (0.78–2.64)	33.8 ± 6.1	115 ± 15	2/12
PDGFR (*n* = 5)	36 (25–56)	1/5	1/5	1.1 (0.8–3.1) [95 (70–270)]	3.23 (1.41–3.33)	33.9 ± 6.4	108 ± 20	0
HER (*n* = 3)	14 (9.8–24)	0	0	1.6 (1.3–2.2) [140 (113–195)]	0.41 (0.40–1.30)	40.5 ± 10.1	109 ± 24	0
BTK (*n* = 1)	5	0	0	2.0 (178)	Negative	42.3	97	0
Total (*n* = 21)	25 (5–41)	1/21	8/21	1.1 (0.8–1.4) [95 (70–123)]	1.83 (0.53–2.90)	35.2 ± 7.3	111 ± 18	2/21

Data are presented as median (range) or *n* (%) or average ± standard deviation.

Alb, serum albumin; Dur., duration; Hb, hemoglobin; mos, months; NS, nephrotic syndrome; SCr, serum creatinine; TCP, thrombocytopenia; U-Pro, proteinuria.

**Table 3:** Clinical features and laboratory parameters of patients at the time of renal biopsy: stratification by serum creatinine trajectory.

Pt	Primary TKI target	Sex	Age (years)	BMI (kg/m²)	TKI-Sx onset (mos)	TKI dur. (mos)	ΔHTN	Edema	AKI	NS	Base SCr mg/dL (μmol/L)	Scr@Bx mg/dL (μmol/L)	Base U-Pro	Peak U-Pro (g/day)	U-Pro@Bx (g/day)	Alb (g/L)	Hb (g/L)	LDH (µ/L)	PLT (×10⁹/L)	uRBC (/μL)	C3 (g/L)
**Recovery group**																					
1	VEGFR	M	55	29.4	91	105	N	Y	N	Y	WNL	1.0 (86)	–	4.61	2.14	28.7	115	200	216	27.5	1.18
2	VEGFR	M	63	24.5	12	24	Y	N	N	Y	WNL	1.2 (108)	2.04	4.04	1.52	30.8	117	220	158	117.1	0.775
3	HER	M	54	23.44	5	5.7	N	N	N	N	WNL	2.8 (251)	–	2.19	2.19	29.6	76	374	482	53	0.88
**Stability group**																					
4	VEGFR	F	48	26.35	0.5	1	Y	Y	N	Y	WNL	0.6 (49)	–	9.28	4.27	29.1	129	286	217	7.9	1.168
5	VEGFR	M	55	31	0.3	0.8	N	N	N	N	WNL	1.2 (102)	–	4.01	2.59	40	136	152	203	1.9	1.65
6	VEGFR	F	53	23.9	0.13	0.6	Y	Y	N	N	WNL	0.6 (55)	–	4.19	0.55	39.4	94	466	42	8.8	1.16
7	VEGFR	F	50	21.16	3	53.4	Y	Y	N	Y	WNL	0.9 (82)	–	3.58	0.49	40.5	106	216	158	12.2	0.123
8	VEGFR	M	51	26.03	9	18	N	Y	N	Y	WNL	0.6 (53)	–	5.59	2.31	21.5	146	295	137	38.9	0.239
9	VEGFR	M	54	23.18	48	53	Y	Y	N	Y	WNL	1.4 (123)	–	8.7	2.8	31.7	96	NA	193	15.4	NA
10	VEGFR	F	27	19.53	16	30	N	N	N	N	WNL	0.6 (57)	3+	2.61	0.94	40	113	211	225	4.6	1.019
11	VEGFR	M	59	17.92	10	26	Y	N	N	N	WNL	1.4 (121)	–	1.48	0.86	29.7	112	178	119	43.4	0.82
12	PDGFR	F	60	28.12	12	59	N	N	N	N	WNL	0.6 (57)	1+	3.3	3.23	38.5	109	193	173	5.1	1.16
13	HER	F	59	20.2	6	34	N	N	N	N	WNL	1.0 (87)	–	0.45	0.39	38.1	120	132	197	2.1	0.769
14	BTK	M	58	26.95	0.5	5	N	N	N	N	WNL	2.0 (178)	–	–	–	42.3	97	312	130	5.5	0.91
**Worsening group**																					
15	PDGFR	F	52	22.5	36	36	N	Y	N	Y	WNL	0.8 (70)	–	7.69	3.99	21.3	113	262	202	71.5	1.16
**Not assessable**																					
16	VEGFR	F	59	23.77	0.5	1	Y	Y	N	N	WNL	0.8 (74)	–	4.62	0.43	42.5	119	185	169	20.9	0.91
17	VEGFR	M	69	23.5	21	41	Y	Y	N	Y	WNL	1.3 (111)	–	8.3	3.44	32.5	101	176	139	22.5	0.84
18	PDGFR	M	44	27.08	0.5	0.5	Y	Y	3	N	10.2 (900)	6.9 (608)	1+	0.42	0.33	35.8	86	339	607	21.4	0.712
19	PDGFR	M	71	21.3	NA	56	Y	Y	N	N	1.2 (110)	3.1 (270)	–	3.86	3.33	35.2	91	186	130	9.4	0.608
20	PDGFR	M	49	26.42	12	25	N	N	N	N	WNL	1.1 (95)	–	3.05	1.41	38.7	143	196	236	6	0.57
21	HER	M	58	27.12	10	14	N	N	N	N	1.2 (103)	1.6 (140)	–	0.41	0.41	54	132	180	188	1.4	1.09

AKI was staged per KDIGO criteria; ‘3’ = Stage 3 AKI, ‘–’ = no AKI.

Proteinuria (U-Pro) values: ‘–’ = negative (dipstick trace negative or <0.15 g/day); quantitative values = 24-h urine protein (g/day); semi-quantitative dipstick: 1+ ≈ 0.3 g/day, 3+ ≈ 3.0 g/day.

@, at; Δ, change; Alb, serum albumin; Base, baseline; Bx, biopsy; Dur., duration; F, female; Hb, hemoglobin; HTN, hypertension; LDH, lactate dehydrogenase; M, male; mos, months; N, no; NA, not available; NS, nephrotic syndrome; PLT, platelet count; Pt, patient; SCr, serum creatinine; Sx, symptoms; uRBC, urinary red blood cells; U-Pro, proteinuria; WNL, within normal limits; Y, yes.

### Pathologic characteristics

**Figure 2:**
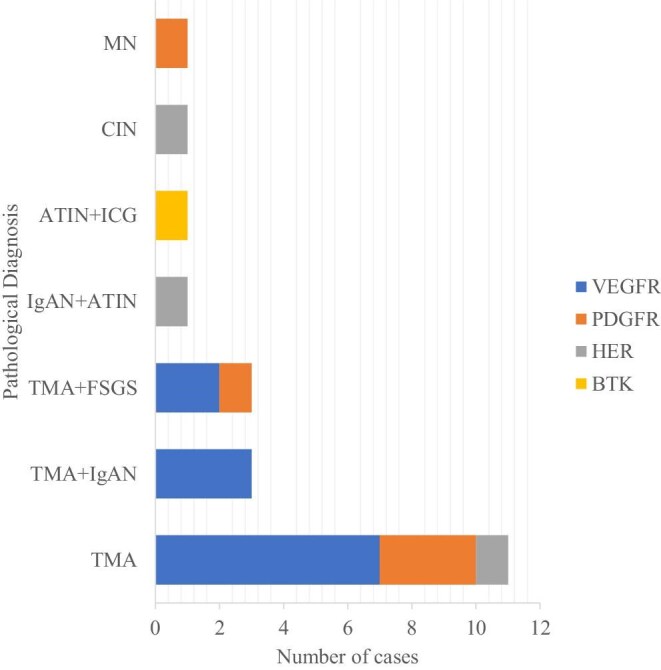
Renal pathological spectrum in 21 patients with TKI-induced nephrotoxicity.

As shown in Fig. 2, thrombotic microangiopathy (TMA)-like lesions constituted 80.9% (17/21) of cases, including isolated TMA (*n* = 11), TMA with IgA nephropathy (IgAN; *n* = 3) and TMA with focal segmental glomerulosclerosis (FSGS) (*n* = 3). Other diagnoses included membranous nephropathy (*n* = 1), IgAN coexisting with acute tubulointerstitial nephritis (*n* = 1), immune-complex glomerulonephritis associated with tubulointerstitial injury (*n* = 1) and chronic interstitial nephritis (*n* = 1).

**Figure 3:**
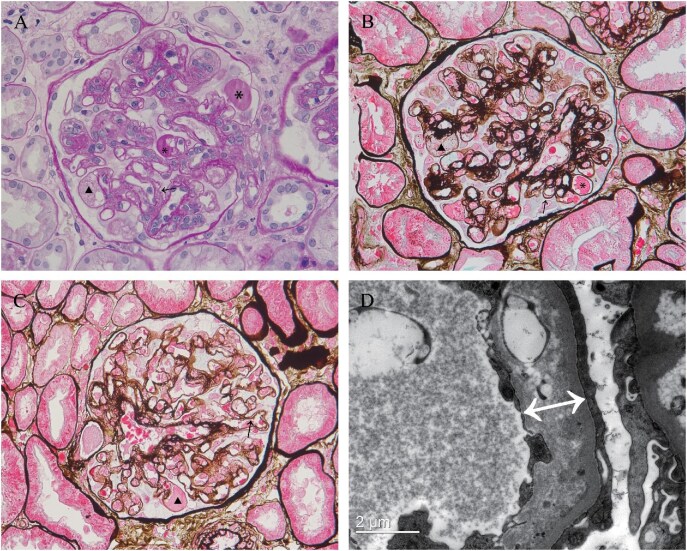
Representative histopathological features of TKI-associated TMA. (**A**–**C**) Light microscopy demonstrates segmental glomerular basement membrane duplication (double contours, upwards arrow), accompanied by subendothelial region dilation with lucent materials (triangles) and hyaline materials (stars). Hyaline material occasionally protrudes into capillary lumina, causing loop narrowing. (D) Electron microscopy reveals widening of the subendothelial space (double-ended arrow). (A) PAS, ×400; (B, C) PASM-Masson, ×400; (D) electron microscopy.

Among the 17 TMA-like cases, histopathology revealed universal segmental glomerular basement membrane duplication (double contours), frequently accompanied by mesangiolysis (76.5%). Additional characteristic findings included subendothelial region dilation with hyaline deposits (58.8%; Fig. 3A and B), capillary loop shrinkage (41.2%) and intracapillary inflammatory infiltrates (35.3%). Variable degrees of chronic injury—including glomerulosclerosis, interstitial fibrosis and vascular sclerosis—were also observed. Notably, VEGFR-TKI-associated cases exhibited distinct pathological patterns: foam cell accumulation (58.8%), ballooning degeneration (50.0%; Fig. 3A–C), endothelial swelling (50.0%) and capillary dilation (50.0%; Fig. 3A–C). Intracapillary thrombi were observed in 17.6% of all TMA cases (Table 4). Immunofluorescence analysis detected mild IgM deposition in 12 cases (70.6%) and C3 deposition in 6 cases (35.3%). Electron microscopy demonstrated diffuse podocyte foot process effacement (100%) and subendothelial widening (76.5%; Fig. 3D) with persistent double contour formation.

**Table 4:** Kidney pathological characteristics of 15 patients with TMA-pattern TKI-associated kidney injury: stratification by serum creatinine trajectory.

	Recovery group		Stability group										Not assessable				
Pt	1	2	4	5	6	7	8	9	10	11	12	13	16	17	18	19	20
Primary TKI target	VEGFR	VEGFR	VEGFR	VEGFR	VEGFR	VEGFR	VEGFR	VEGFR	VEGFR	VEGFR	PDGFR	HER	VEGFR	VEGFR	PDGFR	PDGFR	PDGFR
Path Dx LM	TMA	TMA + IgAN	TMA	TMA	TMA	TMA + IgAN	TMA + FSGS	IgAN + TMA	TMA	TMA	TMA	TMA	TMA	TMA + FSGS	TMA	TMA	TMA + FSGS
**Glom**																	
#Glom	35	25	44	30	34	32	37	15	69	8	28	21	29	31	21	30	35
Glob Scl (%)	3	19	0	10	15	47	3	20	41	0	18	14	10	13	0	70	26
Segm Scl (%)	0	0	14	10	0	3	16	47	10	0	4	0	0	13	0	7	14
Cresc (%)	0	0	5	0	0	0	0	7	0	0	4	0	0	0	0	0	0
Endoth Hypercel.	Y	N	Y	N	Y	Y	Y	Y	N	N	N	N	N	N	N	N	Y
DC	2+	2+	2+	+	2+	2+	2+	2+	2+	+	+	+	+	+	2+	+	+
Mes Hyperpl	2+	+	–	+	+	+	+	+	+	+	+	+	+	+	+	+	+
Mesangiolysis	2+	–	+	+	–	+	2+	+	–	+	+	+	+	+	+	–	+
Cap Dil	Y	Y	N	N	N	Y	N	Y	N	Y	N	N	N	Y	N	N	N
CLS	+	+	–	+	–	–	–	–	+	–	+	–	–	–	+	+	–
Foam cells	Y	Y	Y	N	Y	N	N	Y	N	Y	N	N	N	Y	N	N	N
Hyaline deposits	Y	Y	Y	N	N	Y	Y	Y	Y	Y	N	N	N	Y	N	N	Y
Balloon	Y	Y	N	N	N	Y	N	Y	Y	N	Y	N	N	Y	N	N	N
ICII	+	–	–	–	+	+	–	+	–	–	–	–	+	–	+	–	–
ICT	+	–	+	–	–	–	–	–	–	–	–	–	–	–	–	+	–
**Art**																	
Thromb	N	N	N	N	N	N	Y	Y	N	N	N	N	N	N	Y	N	N
**TI**																	
ATI	+	+	+	+	+	+	+	–	+	+	–	–	+	+	+	+	+
IF/TA	+	+	–	+	+	+	+	+	+	+	+	+	+	+	3+	2+	+
**IF**																	
IgG+	–	2+	+	–	–	+	–	–	–	–	–	–	–	–	–	–	–
IgA+	–	2+	+	–	+–2+	2+	–∼+	2+	–	–	–	–	–	–	+	–	–
IgM+	+	+	+	+	+	–	+	+	+	–	–	–	+	–∼+	+	+	–
C3+	–	+	–	–	+	+	–	+	–	–	–	–	–∼+	–	–	+	–
C1q+	–	–	+	–	–	–	–	–	–	–	–	–	–	–	–	–	–
**EM**																	
Subendo DDs	Y	Y	Y	N	Y	N	Y	Y	N	Y	N	NA	Y	Y	N	Y	N
Mes DDs	Y	Y	Y	N	Y	Y	Y	Y	Y	Y	N	NA	Y	Y	N	Y	Y
Subepi DDs	N	Y	Y	N	N	N	N	N	N	N	N	NA	N	Y	N	N	N
FPE	2+	+	+	+	+	+	2+	+	+	+	+	NA	+	+	+	2+	+
Subendo Wid	Y	Y	Y	Y	Y	N	N	Y	Y	Y	N	NA	Y	Y	Y	Y	Y

Grading symbols: + (positivity), # (number), (%) (percentage); glomerular lesions (double contours/mesangiolysis/FPE): – (<5%), + (5%–50%), 2+ (>50%); tubulointerstitial lesions: – (<5%), + (5%–25%), 2+ (26%–50%), 3+ (>50%); immunofluorescence: – (negative), 1+ (weak), 2+ (moderate), 3+ (strong), range (e.g. 1+ to 3+); FPE: 0 (<10%), 1+ (10%–50%), 2+ (>50%).

Art, arterioles; ATI, acute tubular injury; Balloon, ballooning; Bx SCr, serum creatinine at renal biopsy; Cap Dil, capillary dilation; CLS, capillary loop shrinkage; Cresc, crescents; DDs, electron-dense deposits; EM, electron microscopy; Endoth Hypercell, endothelial hypercellularity/swelling; FPE, foot process effacement; Glob Scl, globally sclerosed; ICII, intracapillary inflammatory infiltrate; ICT, intracapillary thrombi; IF, immunofluorescence; IF/TA, interstitial fibrosis/tubular atrophy; LM, light microscopy; Mes Hyperpl, mesangial hyperplasia; mos, months; NA, not available; N, no; Path Dx, pathological diagnosis; PD, progressive disease; Pt, patient; Segm Scl, segmental sclerosis; Subendo, subendothelial; Subepi, subepithelial; Thromb, thrombosis; TI, tubulointerstitium; Y, yes.

### Treatment and outcomes

Among 18 patients with effective follow-up data, TKI management strategies included treatment discontinuation (*n* = 8), agent substitution (*n* = 5) or continued administration (*n* = 5). Regarding renoprotective strategies, 13 patients received angiotensin-converting enzyme inhibitors/angiotensin receptor blockers (ACEI/ARB) therapy, whereas 5 patients were initiated on immunosuppressive therapy for significant proteinuria (>1 g/day) and/or renal impairment, guided by the finding of immune complex deposition on renal biopsy. At a median follow-up of 9.5 months (IQR 3.0–29.5), proteinuria remission occurred in 55% of cases (CR: *n* = 5; PR: *n* = 5). All patients maintained stable renal function, with 3 showing recovery and 11 maintaining stable serum creatinine levels; none experienced doubling of serum creatinine or progression to end-stage renal disease. No major adverse events attributable to immunosuppressive therapy were observed. Four patients died from cancer progression (Table 5).

**Table 5:** Treatment and outcomes of 21 patients with TKI-associated renal injury: stratification by serum creatinine trajectory.

Pt	Primary TKI target	TKI D/C	Alt TKI	ACEI/ARB	IS therapy	FU (mos)	Bx SCr mg/dL (μmol/L)	FU SCr mg/dL (μmol/L)	Peak U-Pro (g/day)	FU U-Pro (g/day)	U-Pro Rem	Renal survival	Tumor status
Recovery group													
1	VEGFR	Y	N	Y	N	43	1.0 (86)	0.7 (59)	4.61	2.03	PR	CKD 2	CR
2	VEGFR	N	N	Y	Pred	13	1.2 (108)	0.8 (69)	4.04	7.47	NR	Death	PD
3	HER	Y	Ametinib → crizotinib	N	TG	3	2.8 (251)	1.9 (171)	2.19	0.5	PR	CKD 3	SD
**Stability group**													
4	VEGFR	Y	N	Y	N	40	0.6 (49)	0.5 (44)	9.28	0.35	CR	Death	PD
5	VEGFR	N	N	Y	TG	21	1.2 (102)	1.3 (115)	4.01	0.7	CR	Death	PD
6	VEGFR	Y	N	Y	N	0	0.6 (55)	0.6 (53)	4.19	0.24	CR	CKD 3	PD
7	VEGFR	Y	Lenvatinib → donafenib	Y	N	10	0.9 (82)	0.9 (79)	3.58	0.32	CR	CKD 2	SD
8	VEGFR	Y	Lenvatinib → regorafenib	Y	N	9	0.6 (53)	0.7 (59)	5.59	3.26	NR	CKD 1	PR
9	VEGFR	Y	N	Y	N	5.5	1.4 (123)	1.4 (127)	8.7	2.17	PR	CKD 3	SD
10	VEGFR	Y	Regorafenib → Olegbatinib	N	N	44	0.6 (57)	0.6 (57)	2.61	0.94	PR	CKD 1	PD
11	VEGFR	N	N	Y	N	1	1.4 (121)	1.4 (125)	0.86	2.09	NR	CKD 3	SD
12	PDGFR	Y	Dasatinib → flumatinib	Y	N	26	0.6 (57)	0.7 (57)	3.3	1.7	PR	CKD 1	PR
13	HER	Y	N	Y	N	1	1.0 (87)	0.9 (75)	0.45	0.3	NA	CKD 2	SD
14	BTK	Y	N	N	Pred	3.5	2.0 (178)	1.9 (171)	Negative	0.27	NA	CKD 3	SD
**Worsening group**													
15	PDGFR	Y	N	Y	Tac	41	0.8 (70)	1.0 (88)	7.69	0.1	CR	CKD 2	PR
**Not assessable**													
16	VEGFR	Y	N	Y	N	NA	0.8 (74)	NA	4.62	NA	NA	NA	NA
18	PDGFR	N	N	N	N	3	6.9 (608)	NA	0.42	NA	NA	Death	PD
19	PDGFR	Y	N	N	N	NA	3.1 (270)	NA	3.86	NA	NA	NA	NA

Alt TKI, alternative tyrosine kinase inhibitor; Bx SCr, serum creatinine at renal biopsy; CKD, chronic kidney disease; D/C, discontinuation; FU, follow-up; IS therapy, immunosuppressant therapy; mos, months; NA, not available; N, no; NR, no remission; PD, progressive disease; Pred, prednisone; Pt, patient; SCr, serum creatinine; SD, stable disease; Tac, tacrolimus; TG, *Tripterygium wilfordii* glycosides; U-Pro, proteinuria; U-Pro Rem, proteinuria remission.

## DISCUSSION

Our study characterizes the clinicopathological spectrum of TKI-associated renal injury in 21 biopsy-confirmed cases. TMA-like lesions represented the predominant histopathological pattern (80.9%), followed by acute tubulointerstitial nephritis and membranous nephropathy. Despite the limited cohort size, the high prevalence of TMA suggests endothelial injury as a primary mechanism of TKI nephrotoxicity, providing critical insights for clinical management. Robust causality was established for all cases, evidenced by: (i) temporal correlation between TKI initiation and renal dysfunction; (ii) histological concordance with established drug-specific injury patterns [8]; and (iii) systematic exclusion of alternative etiologies (e.g. tumor progression, comorbidities) through comprehensive clinical evaluation. These findings confirm TKI therapy as the primary etiology of renal injury in this cohort.

TMA was the predominant renal lesion in TKI-associated nephrotoxicity, particularly among patients receiving VEGFR- and Breakpoint cluster region-Abelson inhibitors. The characteristic clinical triad comprised nephrotic-range proteinuria, hypertension and histological evidence of chronic endothelial injury (manifest as double contours and segmental glomerulosclerosis), with IgM-dominant subendothelial deposits and ultrastructural mesangial interposition. These findings align with previous reports of TMA induced by lenvatinib [9–13], regorafenib [14] and dasatinib [15, 16]. In contrast to Izzedine *et al*. [17], who reported minimal change disease and FSGS as primary lesions in biopsies performed 2–12 weeks post-initiation, our cohort manifested TMA as the dominant pathology after a median TKI exposure of 25 months (range 5–41). This phenotypic divergence may reflect duration-dependent toxicity. Current models suggest that during early TKI exposure triggers compensatory pro-angiogenic factors through aberrant endothelial–podocyte crosstalk, temporarily preserving glomerular homeostasis [18]. Our findings likely represent advanced endothelial injury from sustained TKI exposure. Mechanistically, TKI-mediated anti-angiogenic effects drive TMA through tripartite pathways: (i) direct VEGF signaling blockade by VEGFR-TKIs compromises endothelial integrity [19]; (ii) EGFR inhibitors indirectly impair dysfunction via VEGF downregulation [20]; and (iii) PDGFR inhibitors impair microvascular integrity through Src kinase-mediated endothelial repair suppression [21] and pericyte dysfunction [22], collectively creating a pro-thrombotic environment conducive to TMA development.

TKI-associated TMA shares clinicopathological features with anti-VEGF monoclonal antibody-induced TMA, including capillary hyalinosis, ballooning degeneration and microaneurysms coexisting with classical endothelial lesions [23]. However, TKI-associated cases demonstrated significantly lower prevalence of these secondary glomerular alterations. This attenuated phenotype may result from partial VEGF signaling preservation through multi-target inhibition, potentially reducing glomerular injury severity relative to selective VEGF blockade. Mechanistically, the broader pharmacological profile of TKIs might ameliorate endothelial stress via compensatory pathways, though experimental validation is needed. Distinct from TMA secondary to atypical hemolytic uremic syndrome or thrombotic thrombocytopenic purpura, TKI-related TMA rarely presents with microthrombi, systemic hematological abnormalities (e.g. thrombocytopenia, hemolytic anemia) or extrarenal involvement. Serum creatinine typically remains stable, likely due to chronic endothelial adaptation and compensatory fibrinolysis rather than acute thrombotic events. The indolent clinical course correlates histologically with predominant chronic TMA features (e.g. double contours, mesangiolysis) rather than acute microvascular occlusion. Collectively, this pathophysiological profile establishes TKI-associated TMA as a distinct drug-induced microangiopathy, characterized by renal-limited, chronic endothelial injury that differs from both antibody-mediated VEGF inhibition and complement/ADAMTS13-driven thrombotic microangiopathies.

The coexistence of IgAN in three biopsy-confirmed TMA cases presents a significant diagnostic challenge. Although the predominantly mesangial IgA distribution suggests potential pre-existing or concurrent IgAN—the most prevalent primary glomerulonephritis—subendothelial immunoglobulin deposition could be potentiated by TKI-induced endothelial hyperpermeability [23]. Given the high background prevalence of IgAN, its independent occurrence cannot be fully excluded. Recent data implicate endothelial dysfunction in IgAN progression [24], warranting further studies to clarify potential shared pathways between TKI nephrotoxicity and IgAN pathogenesis. Acute tubulointerstitial nephritis (ATIN) represented another distinct injury pattern, invariably co-occurring with immune-complex glomerulonephritis. This differs markedly from classical drug-induced ATIN mediated by type IV hypersensitivity [25], suggesting TKI-specific immunomodulatory mechanisms that merit in-depth mechanistic investigation.

Clinical management should prioritize strict hypertension control and proteinuria reduction through first-line ACEI/ARB therapy, reserving immunosuppression for cases with concurrent immune-mediated glomerulopathy. The documented reversibility of early-stage glomerular injury [16, 18] underscores the critical importance of prompt TKI discontinuation when renal dysfunction is detected. For patients requiring continuous oncological control, substitution with alternative TKIs exhibiting lower nephrotoxicity profiles offers a viable alternative. Pretreatment genetic screening (e.g. CFH variants [26], ADAMTS13 activity [27] should be integrated into risk stratification and enhanced monitoring protocols. Overall renal prognosis following discontinuation in this cohort was favorable.

Several limitations warrant acknowledgment. First, residual confounding may persist from prior anti-VEGF antibody exposure in four cases, despite rigorous clinical assessment. Second, the limited cohort size (*n* = 21) restricts statistical power for subgroup analyses and external validity. Third, the absence of molecular profiling or genetic screening precludes mechanistic interrogation of TKI-associated nephrotoxicity. Future multicenter studies with larger cohorts should employ integrated multi-omics approaches to elucidate pathogenic pathways, while implementing longitudinal assessments of TKI-induced renal injury across diverse populations to improve clinical applicability.

In summary, TKI therapy predominantly induces renal-limited TMA through endothelial injury mediated by anti-angiogenic effects. The characteristic clinical triad—hypertension, nephrotic-range proteinuria and preserved renal function—typically demonstrates reversibility following drug cessation combined with supportive care. These observations underscore the necessity for rigorous renal monitoring during TKI administration and evidence-based risk-benefit evaluation in oncological management.

## Supplementary Material

sfaf394_Supplemental_File

## Data Availability

The data underlying this article cannot be shared publicly due to the privacy of individuals that participated in the study. The de-identified data will be shared on reasonable request to the corresponding author.
