# Supplementary material for: Biopsy-proven tyrosine kinase inhibitor–associated renal injury: a case series
Source: Clin Kidney J. 2025 Dec 13;19(1):sfaf394. doi: 10.1093/ckj/sfaf394 (PMC12780777; doi:10.1093/ckj/sfaf394)
Supplement: sfaf394_Supplemental_File [file sfaf394_supplemental_file.docx]

**Table S1. Evidence Levels for the Association Between Different Classes of TKIs and Renal Injury**

| **Evidence level** | **VEGFR Group(n=12)** | **PDGFR Group(n=5)** | **HER Group(n=3)** | **BTK Group（n=1）** | **Total （n=21）** |
| --- | --- | --- | --- | --- | --- |
| Certain, n | 1 | 0 | 0 | 0 | 1 |
| Probable, n | 10 | 4 | 3 | 1 | 18 |
| Possible, n | 1 | 1 | 0 | 0 | 2 |

Abbreviations: VEGFR Vascular Endothelial Growth Factor Receptor, PDGFR Platelet-Derived Growth Factor Receptor, HER Human Epidermal Growth Factor Receptor, BTK Bruton's Tyrosine Kinase.
